# An incremental mirror descent subgradient algorithm with random sweeping and proximal step

**DOI:** 10.1080/02331934.2018.1482491

**Published:** 2018-06-14

**Authors:** Radu Ioan Boţ, Axel Böhm

**Affiliations:** Faculty of Mathematics, University of Vienna, Vienna, Austria

**Keywords:** Nonsmooth convex minimization, incremental mirror descent algorithm, global rate of convergence, random sweeping, 90C25, 90C90, 90C06

## Abstract

We investigate the convergence properties of incremental mirror descent type subgradient algorithms for minimizing the sum of convex functions. In each step, we only evaluate the subgradient of a single component function and *mirror* it back to the feasible domain, which makes iterations very cheap to compute. The analysis is made for a randomized selection of the component functions, which yields the deterministic algorithm as a special case. Under supplementary differentiability assumptions on the function which induces the mirror map, we are also able to deal with the presence of another term in the objective function, which is evaluated via a proximal type step. In both cases, we derive convergence rates of $O(1/\sqrt{k})$ in expectation for the *k*th best objective function value and illustrate our theoretical findings by numerical experiments in positron emission tomography and machine learning.

## 1. Introduction

We consider the problem of minimizing the sum of nonsmooth convex functions

(1) $$\min_{x\in C}\sum_{i=1}^{m}f_{i}(x),$$

where $C\subseteq R^{n}$ is a nonempty, convex and closed set and, for every i=1,…,m, the so-called *component functions*$f_{i}:R^{n}\to \bar{R}:=R\cup \{\pm \infty \}$ are assumed to be proper and convex and will be evaluated via their respective subgradients. Implicitly we will assume that *m* is *large* and it is therefore very costly to evaluate all component functions in each iteration. Consequently, we will examine algorithms which only use the subgradient of a single component function in each iteration. These so-called *incremental algorithms*, see [1,2], have been applied for large-scale problems arising in tomography [3], generalized assignment problems [2] or machine learning [4]. We refer also to [5] for a slightly different approach, where in the spirit of incremental algorithms only the gradient of one of the component functions is evaluated in each step, but gradients at old iterates are used for the other components. Both subgradient algorithms and incremental methods usually require decreasing stepsizes in order to converge, which makes them slow near an optimal solution. However, they provide a very small number of iterations a low accuracy optimal value and possess a rate of convergence which is almost independent of the dimension of the problem. We refer the reader to [6] for a subgradient algorithm designed for the minimization of a nonsmooth nonconvex function under the making use of proximal subgradients.

When solving optimization problems of type (1), one might want to capture in the formulation of the iterative scheme the geometry of the feasible set *C*. This can be done by a so-called *mirror map*, that mirrors each iterate onto the feasible set. The Bregman distance associated with the function that induces the mirror map plays an essential role in the convergence analysis and in the formulation of convergence rates results (see [7,8]). So-called *mirror descent algorithms* were first discussed in [9] and more recently in [8,10,11] in a very general framework, in [4,12] from a statistical learning point of view and in [13] for the case of dynamical systems. The mirror map can be viewed as a generalization of the ordinary orthogonal projection on *C* in Hilbert spaces (see Example 2.4), but allows also for a more careful consideration of the structure of the problems, as it is the case when the objective function is subdifferentiable only on the relative interior of the feasible set. In such a setting, one can design a mirror map which maps not onto the entire feasible set but only on a subset of it where the objective function is subdifferentiable (see Example 2.5).

There exists already a rich literature on incremental algorithms dealing with similar problems. In [1,2] incremental subgradient methods with a random selection of the component functions and even projections onto a feasible set are considered, but no mirror descent. Incremental subgradient algorithms utilizing mirror descent techniques are investigated in [3]; however, an additional projection onto the feasible set is required which thus excludes the case where domf⊉C (this is taken care of in our case by the weak assumption that $\text{im}(\nabla H^{*})\subseteq \mathrm{dom}\;f$). Furthermore, the results appearing in Section 4 discussing Bregman proximal steps appear to completely novel for this kind of problems and are only known from a forward–backward setting [7].

The basic concepts in the formulation of mirror descent algorithms are recalled in Section 2. We also provide some illustrating examples, which present some special cases, as the general setting might not be immediately intuitive. In Section 3 we formulate an incremental mirror descent subgradient algorithm with random sweeping of the component functions which we show to have a convergence rate of $O(1/\sqrt{k})$ in expectation for the *k*th best objective function value. In Section 4 we ask additionally for differentiability of the function which induces the mirror map and are then able to add another nonsmooth convex function to the objective function which is evaluated in the iterative scheme by a proximal type step. For the resulting algorithm, we show a similar convergence result. In the last section, we illustrate the theoretical findings by numerical experiments in positron emission tomography (PET) and machine learning.

## 2. Elements of convex analysis and the mirror descent algorithm

Throughout the paper, we assume that $R^{n}$ is endowed with the Euclidean inner product ⟨⋅,⋅⟩ and corresponding norm $∥\cdot ∥=\sqrt{\left\langle \cdot ,\cdot \right\rangle }$. For a nonempty convex set $C\subseteq R^{n}$ we denote by riC its *relative interior*, which is the interior of *C* relative to its affine hull. For a convex function $f:R^{n}\to \bar{R}$ we denote by $\mathrm{dom}\;f:=\{x\in R^{n}:f(x)<+\infty \}$ its *effective domain* and say that *f* is *proper*, if f>−∞ and domf≠∅. The subdifferential of *f* at $x\in R^{n}$ is defined as $\partial f(x):=\{p\in R^{n}:f(y)\geq f(x)+\langle p,y-x\rangle \forall y\in R^{n}\}$, for f(x)∈R, and as ∂f(x):=∅, otherwise. We will write $f^{\prime }(x)$ for an arbitrary subgradient, i.e. an element of the subdifferential ∂f(x).

Problem 2.1 Consider the optimization problem

(2) $$\min_{x\in C}f(x),$$

where $C\subseteq R^{n}$ is a nonempty, convex and closed set, $f:R^{n}\to \bar{R}$ is a proper and convex function, and $H:R^{n}\to \bar{R}$ is a proper, lower semicontinuous and *σ*-strongly convex function such that $C=\bar{\mathrm{dom}\;H}$ and $\text{im}(\nabla H^{*})\subseteq \mathrm{int}(\mathrm{dom}\;f)$.

We say that $H:R^{n}\to \bar{R}$ is *σ*-strongly convex for σ>0, if for every $x,x^{\prime }\in R^{n}$ and every λ∈[0,1] it holds $\left(\sigma /2)\lambda (1-\lambda )∥x-x^{\prime }{∥}^{2}+H(\lambda x+(1-\lambda )x^{\prime })\leq \lambda H(x)+(1-\lambda )H(x^{\prime }\right)$. It is well known that, when *H* is proper, lower semicontinuous and *σ*-strongly convex, then its *conjugate function*$H^{*}:R^{n}\to \bar{R},H^{*}(y)=\underset{x\in R^{n}}{\sup}\{\langle y,x\rangle -H(x)\}$, is Fréchet differentiable (thus it has full domain) and its gradient $\nabla H^{*}$ is 1/σ-Lipschitz continuous or, equivalently, $H^{*}$ is Fréchet differentiable and its gradient $\nabla H^{*}$ is *σ*-cocoercive, which means that for every $y,y^{\prime }\in R^{n}$ it holds $\sigma ∥\nabla H^{*}(y)-\nabla H^{*}(y^{\prime }){∥}^{2}\leq \langle y-y^{\prime },\nabla H^{*}(y)-\nabla H^{*}(y^{\prime })\rangle$. Recall that $\text{im}(\nabla H^{*}):=\{\nabla H^{*}(y):y\in R^{n}\}$.

The following mirror descent algorithm has been introduced in [10] under the name *dual averaging*.

Algorithm 2.2 Consider for some initial values $x_{0}\in \mathrm{int}(\mathrm{dom}\;f),y_{0}\in R^{n}$ and sequence of positive stepsizes $(t_{k}{)}_{k\geq 0}$ the following iterative scheme:

$$(\forall \;k\geq 0)\;\left[\begin{array}{l} y_{k+1}=y_{k}-t_{k}f^{\prime }(x_{k})\\ x_{k+1}=\nabla H^{*}(y_{k+1}). \end{array}\right.$$

We notice that, since the sequence $(x_{k}{)}_{k\geq 0}$ is contained in the interior of the effective domain of *f*, the algorithm is well defined. The assumptions concerning the function *H*, which induces the mirror map $\nabla H^{*}$, are not consistent in the literature. Sometimes *H* is assumed to be a *Legendre function* as in [7], or strongly convex and differentiable as in [8,11]. In the following section, we will only assume that *H* is proper, lower semicontinuous and strongly convex.

Example 2.3 For $H=\frac{1}{2}∥\cdot {∥}^{2}$ we have that $H^{*}=\frac{1}{2}∥\cdot {∥}^{2}$ and thus $\nabla H^{*}$ is the identity operator on $R^{n}$. Consequently, Algorithm 2.2 reduces to the classical subgradient method:

$$(\forall \;k\geq 0)\;x_{k+1}=x_{k}-t_{k}f^{\prime }(x_{k}).$$

Example 2.4 For $C\subseteq R^{n}$ a nonempty, convex and closed set, take $H(x)=\frac{1}{2}∥x{∥}^{2}$, for x∈C, and H(x)=+∞, otherwise. Then $\nabla H^{*}=P_{C}$, where $P_{C}$ denotes the orthogonal projection onto *C*. In this setting, Algorithm 2.2 becomes:

$$(\forall \;k\geq 0)\;\left[\begin{array}{l} y_{k+1}=y_{k}-t_{k}f^{\prime }(x_{k})\\ x_{k+1}=P_{C}(y_{k+1}). \end{array}\right.$$

This iterative scheme is similar to, but different from the well-known subgradient projection algorithm, which reads:

$$(\forall \;k\geq 0)\;\left[\begin{array}{l} y_{k+1}=x_{k}-t_{k}f^{\prime }(x_{k})\\ x_{k+1}=P_{C}(y_{k+1}). \end{array}\right.$$

Example 2.5 When considering numerical experiments in PET, one often minimizes over the unit simplex $\Delta :=\{x=(x_{1},\ldots ,x_{n}{)}^{T}\in R^{n}:\sum_{j=1}^{n}x_{j}=1,x\geq 0\}$. An appropriate choice for the function *H* is $H(x)=\sum_{j=1}^{n}x_{j}\log(x_{j})$ for x∈Δ, where 0log⁡(0)=0, and H(x)=+∞, if x∉Δ. In this case $\nabla H^{*}$ is given for every $y\in R^{n}$ by

$$\nabla H^{*}(y)=\frac{1}{\sum_{i=1}^{n}\exp(y_{i})}(\exp(y_{1}),\exp(y_{2}),\ldots ,\exp(y_{n}){)}^{T},$$

and maps into the relative interior of Δ.

The following result will play an important role in the convergence analysis that we will carry out in the next sections.

Lemma 2.6 Let $H:R^{n}\to \bar{R}$ be a proper, lower semicontinuous and *σ*-strongly convex function, for σ>0, $x\in R^{n}$ and y∈∂H(x). Then it holds

$$H(x)+\langle y,x^{\prime }-x\rangle +\frac{\sigma }{2}∥x^{\prime }-x{∥}^{2}\leq H(x^{\prime })\;\forall x^{\prime }\in R^{n}.$$

Proof. The function $\tilde{H}(\cdot ):=H(\cdot )-(\sigma /2)∥\cdot {∥}^{2}$ is convex and $y-\sigma x\in \partial \tilde{H}(x)$. Thus

$$\tilde{H}(x)+\langle y-\sigma x,\tilde{x}-x\rangle \leq \tilde{H}(\tilde{x})\;\forall \;\tilde{x}\in R^{n}$$

or, equivalently,

$$H(x)-\frac{\sigma }{2}∥x{∥}^{2}+\langle y-\sigma x,\tilde{x}-x\rangle \leq H(\tilde{x})-\frac{\sigma }{2}∥\tilde{x}{∥}^{2}\;\forall \;\tilde{x}\in R^{n}.$$

Rearranging the terms, leads to the desired conclusion.

## 3. A stochastic incremental mirror descent algorithm

In this section, we will address the following optimization problem.

Problem 3.1 Consider the optimization problem

(3) $$\min_{x\in C}\sum_{i=1}^{m}f_{i}(x),$$

where $C\subseteq R^{n}$ is a nonempty, convex and closed set, for every i=1,…,m, the functions $f_{i}:R^{n}\mapsto \bar{R}$ are proper and convex, and $H:R^{n}\to \bar{R}$ is a proper, lower semicontinuous and *σ*-strongly convex function such that $C=\bar{\mathrm{dom}\;H}$ and $\text{im}(\nabla H^{*})\subseteq \mathrm{int}(\overset{m}{\underset{i=1}{⋂}}\mathrm{dom}\;f_{i})$.

In this section, we apply the dual averaging approach described in Algorithm 2.2 to the optimization problem (3) by only using the subgradient of a component function at a time. This *incremental* approach (see, also, [1,2]) is similar to but slightly different from the extension suggested in [8]. Furthermore, we introduce a stochastic sweeping of the component functions. For a similar strategy, but in the random selection of coordinates we refer to [14].

Algorithm 3.2 Consider for some initial values $x_{0}\in \mathrm{int}(\overset{m}{\underset{i=1}{⋂}}\mathrm{dom}\;f_{i}),y_{m,-1}\in R^{n}$ and sequence of positive stepsizes $(t_{k}{)}_{k\geq 0}$ the following iterative scheme:

$$(\forall \;k\geq 0)\;\left[\begin{array}{l} \psi_{0,k}=x_{k}\\ y_{0,k}=y_{m,k-1}\\ for\;i=1,\ldots ,m\\ \;\;y_{i,k}=y_{i-1,k}-\epsilon_{i,k}\frac{t_{k}}{p_{i}}f_{i}^{\prime }(\psi_{i-1,k})\\ \;\;\psi_{i,k}=\nabla H^{*}(y_{i,k})\\ end\\ x_{k+1}=\psi_{m,k}, \end{array}\right.$$

where $\epsilon_{i,k}isa\{0,1\}$ valued random variable for every i=1,…,m and k≥0, such that $\epsilon_{i,k}$ is independent of $\psi_{i-1,k}$ and $P(\epsilon_{i,k}=1)=p_{i}$ for every i=1,…,m and k≥0.

One can notice that in the above iterative scheme $y_{i,k}\in \partial H(\psi_{i,k})$ for every i=1,…,m and k≥0.

In the convergence analysis of Algorithm 3.2 we will make use of the following *Bregman-distance-like function* associated to the proper and convex function $H:R^{n}\to \bar{R}$ and defined as

(4) $$d_{H}:R^{n}\times \mathrm{dom}\;H\times R^{n}\to \bar{R},\;d_{H}(x,y,z):=H(x)-H(y)-\langle z,x-y\rangle .$$

We notice that $d_{H}(x,y,z)\geq 0$ for every $(x,y)\in R^{n}\times \mathrm{dom}\;H$ and every z∈∂H(y), due to subgradient inequality.

The function $d_{H}$ is an extension of the *Bregman distance* (see [4,7,11]), which is associated to a proper and convex function $H:R^{n}\to \bar{R}$ fulfilling $\mathrm{dom}\;\nabla H:=\{x\in R^{n}:H\;\text{is differentiable at}\;x\}\neq \emptyset$ and defined as

(5) $$D_{H}:R^{n}\times \mathrm{dom}\;\nabla H\to \bar{R},\;D_{H}(x,y)=H(x)-H(y)-\langle \nabla H(y),x-y\rangle .$$

Theorem 3.3 In the setting of Problem 3.1, assume that the functions $f_{i}$ are $L_{f_{i}}$-Lipschitz continuous on $\mathrm{im}(\nabla H^{*})$ for i=1,…,m. Let $(x_{k}{)}_{k\geq 0}$ be a sequence generated by Algorithm 3.2. Then for every N≥1 and every $y\in R^{n}$ it holds

$$\begin{array}{rl} & E\left(\min_{0\leq k\leq N-1}\sum_{i=1}^{m}f_{i}(x_{k})-\sum_{i=1}^{m}f_{i}(y)\right)\\ & \;\leq \frac{d_{H}(y,x_{0},y_{0,0})+\frac{1}{\sigma }{\left(\sum_{i=1}^{m}L_{f_{i}}\right)}^{2}\left({\left(\sum_{i=1}^{m}\frac{1}{p_{i}^{2}}\right)}^{2}+1\right)\sum_{k=0}^{N-1}t_{k}^{2}}{\sum_{k=0}^{N-1}t_{k}}. \end{array}$$

Proof. Let $y\in \overset{m}{\underset{i=1}{⋂}}\mathrm{dom}\;f_{i}\cap \mathrm{dom}\;H$ be fixed. For *y* outside this set the conclusion follows automatically.

For every i=1,…,m and every k≥0 it holds

$$\begin{array}{rl} d_{H}(y,\psi_{i,k},y_{i,k}) & =H(y)-H(\psi_{i,k})-\langle y_{i,k},y-\psi_{i,k}\rangle \\ & =H(y)-H(\psi_{i,k})-\left\langle y_{i-1,k}-\frac{t_{k}}{p_{i}}\epsilon_{i,k}f_{i}^{\prime }(\psi_{i-1,k}),y-\psi_{i,k}\right\rangle . \end{array}$$

Rearranging the terms, this yields for every i=1,…,m and every k≥0 to

$$\begin{array}{rl} d_{H}(y,\psi_{i,k},y_{i,k}) & =d_{H}(y,\psi_{i-1,k},y_{i-1,k})-d_{H}(\psi_{i,k},\psi_{i-1,k},y_{i-1,k})+\frac{t_{k}}{p_{i}}\epsilon_{i,k}\langle f_{i}^{\prime }(\psi_{i-1,k}),y-\psi_{i,k}\rangle \\ & =d_{H}(y,\psi_{i-1,k},y_{i-1,k})-d_{H}(\psi_{i,k},\psi_{i-1,k},y_{i-1,k})+\frac{t_{k}}{p_{i}}\epsilon_{i,k}\langle f_{i}^{\prime }(\psi_{i-1,k}),y-\psi_{i-1,k}\rangle \\ & \;-\frac{t_{k}}{p_{i}}\epsilon_{i,k}\langle f_{i}^{\prime }(\psi_{i-1,k}),\psi_{i,k}-\psi_{i-1,k}\rangle \\ & \leq d_{H}(y,\psi_{i-1,k},y_{i-1,k})-d_{H}(\psi_{i,k},\psi_{i-1,k},y_{i-1,k})+\frac{t_{k}}{p_{i}}\epsilon_{i,k}(f_{i}(y)-f_{i}(\psi_{i-1,k}))\\ & \;+\frac{t_{k}}{p_{i}}\epsilon_{i,k}∥f_{i}^{\prime }(\psi_{i-1,k})∥∥\psi_{i-1,k}-\psi_{i,k}∥. \end{array}$$

From here we get for every i=1,…,m and every k≥0

$$\begin{array}{rl} d_{H}(y,\psi_{i,k},y_{i,k}) & \leq d_{H}(y,\psi_{i-1,k},y_{i-1,k})-d_{H}(\psi_{i,k},\psi_{i-1,k},y_{i-1,k})+\frac{t_{k}}{p_{i}}\epsilon_{i,k}(f_{i}(y)-f_{i}(\psi_{i-1,k}))\\ & \;+\frac{1}{\sigma }t_{k}^{2}\frac{1}{p_{i}^{2}}\epsilon_{i,k}^{2}∥f_{i}^{\prime }(\psi_{i-1,k}){∥}^{2}+\frac{\sigma }{4}∥\psi_{i-1,k}-\psi_{i,k}{∥}^{2} \end{array}$$

which, by using that *H* is *σ*-strongly convex and Lemma 2.6, yields

$$\begin{array}{rl} d_{H}(y,\psi_{i,k},y_{i,k}) & \leq d_{H}(y,\psi_{i-1,k},y_{i-1,k})-d_{H}(\psi_{i,k},\psi_{i-1,k},y_{i-1,k})+\frac{t_{k}}{p_{i}}\epsilon_{i,k}(f_{i}(y)-f_{i}(\psi_{i-1,k}))\\ & \;+\frac{1}{\sigma }t_{k}^{2}\frac{1}{p_{i}^{2}}\epsilon_{i,k}∥f_{i}^{\prime }(\psi_{i-1,k}){∥}^{2}+\frac{1}{2}d_{H}(\psi_{i,k},\psi_{i-1,k},y_{i-1,k})\\ & =d_{H}(y,\psi_{i-1,k},y_{i-1,k})+\frac{t_{k}}{p_{i}}\epsilon_{i,k}(f_{i}(y)-f_{i}(\psi_{i-1,k}))+\frac{1}{\sigma }t_{k}^{2}\frac{1}{p_{i}^{2}}\epsilon_{i,k}∥f_{i}^{\prime }(\psi_{i-1,k}){∥}^{2}\\ & \;-\frac{1}{2}d_{H}(\psi_{i,k},\psi_{i-1,k},y_{i-1,k}). \end{array}$$

Using the fact that $f_{i}$ is $L_{f_{i}}$-Lipschitz continuous, it follows that $∥f_{i}^{\prime }(\psi_{i-1,k})∥\leq L_{f_{i}}$, for every *i*=1,...,*m* and every k≥0, thus

$$\begin{array}{rl} d_{H}(y,\psi_{i,k},y_{i,k}) & \leq d_{H}(y,\psi_{i-1,k},y_{i-1,k})+\frac{t_{k}}{p_{i}}\epsilon_{i,k}(f_{i}(y)-f_{i}(\psi_{i-1,k}))+\frac{1}{\sigma }t_{k}^{2}\frac{1}{p_{i}^{2}}\epsilon_{i,k}L_{f_{i}}^{2}\\ & \;-\frac{1}{2}d_{H}(\psi_{i,k},\psi_{i-1,k},y_{i-1,k}). \end{array}$$

Since all the involved functions are measurable, we can take the expected value on both sides of the above inequality and get due to the assumed independence of $\epsilon_{i,k}$ and $\psi_{i-1,k}$ for every i=1,…,m and every k≥0

$$\begin{array}{rl} E(d_{H}(y,\psi_{i,k},y_{i,k}))\leq & E(d_{H}(y,\psi_{i-1,k},y_{i-1,k}))+E\left(\frac{t_{k}}{p_{i}}(f_{i}(y)-f_{i}(\psi_{i-1,k}))\right)E(\epsilon_{i,k})\\ & \;+\frac{1}{\sigma }t_{k}^{2}\frac{1}{p_{i}^{2}}L_{f_{i}}^{2}E(\epsilon_{i,k})-E\left(\frac{1}{2}d_{H}(\psi_{i,k},\psi_{i-1,k},y_{i-1,k})\right). \end{array}$$

Since $E(\epsilon_{i,k})=p_{i}$, we get for every i=1,…,m and every k≥0

$$\begin{array}{rl} E(d_{H}(y,\psi_{i,k},y_{i,k})) & \leq E(d_{H}(y,\psi_{i-1,k},y_{i-1,k}))+E(t_{k}(f_{i}(y)-f_{i}(\psi_{i-1,k})))\\ & \;+\frac{1}{\sigma }t_{k}^{2}\frac{1}{p_{i}}L_{f_{i}}^{2}-E\left(\frac{1}{2}d_{H}(\psi_{i,k},\psi_{i-1,k},y_{i-1,k})\right). \end{array}$$

Summing the above inequality for i=1,…,m and using that

$$\begin{array}{r} \sum_{i=1}^{m}L_{f_{i}}^{2}\frac{1}{p_{i}}\leq {\left(\sum_{i=1}^{m}L_{f_{i}}^{4}\right)}^{1/2}{\left(\sum_{i=1}^{m}\frac{1}{p_{i}^{2}}\right)}^{1/2}\leq {\left(\sum_{i=1}^{m}L_{f_{i}}\right)}^{2}{\left(\sum_{i=1}^{m}\frac{1}{p_{i}^{2}}\right)}^{1/2}, \end{array}$$

it yields for every k≥0

$$\begin{array}{rl} E(d_{H}(y,\psi_{m,k},y_{m,k})) & \leq E(d_{H}(y,x_{k},y_{0,k}))+E\left(t_{k}\sum_{i=1}^{m}(f_{i}(y)-f_{i}(\psi_{i-1,k}))\right)\\ & \;+\frac{1}{\sigma }t_{k}^{2}{\left(\sum_{i=1}^{m}L_{f_{i}}\right)}^{2}{\left(\sum_{i=1}^{m}\frac{1}{p_{i}^{2}}\right)}^{1/2}-E\left(\sum_{i=1}^{m}\frac{1}{2}d_{H}(\psi_{i,k},\psi_{i-1,k},y_{i-1,k})\right) \end{array}$$

or, equivalently,

$$\begin{array}{rl} E(d_{H}(y,\psi_{m,k},y_{m,k})) & \leq E(d_{H}(y,x_{k},y_{0,k}))+E\left(t_{k}\sum_{i=1}^{m}(f_{i}(y)-f_{i}(x_{k})+f_{i}(x_{k})-f_{i}(\psi_{i-1,k}))\right)\\ & \;+\frac{1}{\sigma }t_{k}^{2}{\left(\sum_{i=1}^{m}L_{f_{i}}\right)}^{2}{\left(\sum_{i=1}^{m}\frac{1}{p_{i}^{2}}\right)}^{1/2}-E\left(\sum_{i=1}^{m}\frac{1}{2}d_{H}(\psi_{i,k},\psi_{i-1,k},y_{i-1,k})\right). \end{array}$$

Thus, for every k≥0,

(6) $$\begin{array}{rl} E(d_{H}(y,\psi_{m,k},y_{m,k})) & \leq E(d_{H}(y,x_{k},y_{0,k}))+t_{k}E\left(\sum_{i=1}^{m}f_{i}(y)-\sum_{i=1}^{m}f_{i}(x_{k})\right)\\ & \;+\frac{1}{\sigma }t_{k}^{2}{\left(\sum_{i=1}^{m}L_{f_{i}}\right)}^{2}{\left(\sum_{i=1}^{m}\frac{1}{p_{i}^{2}}\right)}^{1/2}-E\left(\sum_{i=1}^{m}\frac{1}{2}d_{H}(\psi_{i,k},\psi_{i-1,k},y_{i-1,k})\right)\\ & \;+E\left(t_{k}\sum_{i=1}^{m}(f_{i}(x_{k})-f_{i}(\psi_{i-1,k}))\right). \end{array}$$

On the other hand, by using the Lipschitz continuity of $\nabla H^{*}$ it yields for every k≥0

$$\begin{array}{rl} \sum_{i=1}^{m}(f_{i}(x_{k})-f_{i}(\psi_{i-1,k})) & =\sum_{i=2}^{m}\sum_{j=1}^{i-1}(f_{i}(\psi_{j-1,k})-f_{i}(\psi_{j,k}))\\ & \leq \sum_{i=2}^{m}\sum_{j=1}^{i-1}L_{f_{i}}∥\psi_{j-1,k}-\psi_{j,k}∥\leq \left(\sum_{l=1}^{m}L_{f_{l}}\right)\sum_{i=2}^{m}∥\psi_{i-1,k}-\psi_{i,k}∥,\\ & \leq \left(\sum_{l=1}^{m}L_{f_{l}}\right)\sum_{i=2}^{m}∥\nabla H^{*}(y_{i-1,k})-\nabla H^{*}(y_{i,k})∥\\ & \leq \frac{1}{\sigma }\left(\sum_{l=1}^{m}L_{f_{l}}\right)\sum_{i=2}^{m}∥y_{i-1,k}-y_{i,k}∥\\ & =\frac{1}{\sigma }\left(\sum_{l=1}^{m}L_{f_{l}}\right)\sum_{i=2}^{m}∥\epsilon_{i,k}\frac{t_{k}}{p_{i}}f_{i}^{\prime }(\psi_{i-1,k})∥\\ & \leq \frac{1}{\sigma }t_{k}\left(\sum_{l=1}^{m}L_{f_{l}}\right)\left(\sum_{i=1}^{m}\frac{\epsilon_{i,k}}{p_{i}}L_{f_{i}}\right). \end{array}$$

Therefore, for every k≥0

(7) $$\begin{array}{rl} E\left(t_{k}\sum_{i=1}^{m}(f_{i}(x_{k})-f_{i}(\psi_{i-1,k}))\right) & \leq \frac{1}{\sigma }t_{k}^{2}\left(\sum_{l=1}^{m}L_{f_{l}}\right)E\left(\sum_{i=1}^{m}\frac{\epsilon_{i,k}}{p_{i}}L_{f_{i}}\right)\\ & \leq \frac{1}{\sigma }t_{k}^{2}{\left(\sum_{i=1}^{m}L_{f_{i}}\right)}^{2}. \end{array}$$

Combining (6) and (7) gives for every k≥0

(8) $$\begin{array}{rl} E(d_{H}(y,\psi_{m,k},y_{m,k})) & \leq E(d_{H}(y,x_{k},y_{0,k}))+t_{k}E\left(\sum_{i=1}^{m}f_{i}(y)-\sum_{i=1}^{m}f_{i}(x_{k})\right)\\ & \;+\frac{1}{\sigma }t_{k}^{2}{\left(\sum_{i=1}^{m}L_{f_{i}}\right)}^{2}{\left(\sum_{i=1}^{m}\frac{1}{p_{i}^{2}}\right)}^{1/2}-E\left(\sum_{i=1}^{m}\frac{1}{2}d_{H}(\psi_{i,k},\psi_{i-1,k},y_{i-1,k})\right)\\ & \;+\frac{1}{\sigma }t_{k}^{2}{\left(\sum_{i=1}^{m}L_{f_{i}}\right)}^{2}. \end{array}$$

Since $\psi_{m,k}=x_{k+1}$ and $y_{m,k}=y_{0,k+1}$ we get for every k≥0 that

$$\begin{array}{rl} E(d_{H}(y,x_{k+1},y_{0,k+1})) & \leq E(d_{H}(y,x_{k},y_{0,k}))+t_{k}E\left(\sum_{i=1}^{m}f_{i}(y)-\sum_{i=1}^{m}f_{i}(x_{k})\right)\\ & \;+\frac{1}{\sigma }t_{k}^{2}{\left(\sum_{i=1}^{m}L_{f_{i}}\right)}^{2}\left({\left(\sum_{i=1}^{m}\frac{1}{p_{i}^{2}}\right)}^{2}+1\right). \end{array}$$

By summing up this inequality from *k*=0 to *N*−1, where N≥1, we get

$$\begin{array}{rl} & \sum_{k=0}^{N-1}t_{k}E\left(\sum_{i=1}^{m}f_{i}(x_{k})-\sum_{i=1}^{m}f_{i}(y)\right)+E(d_{H}(y,x_{N},y_{0,N}))\\ & \leq E(d_{H}(y,x_{0},y_{0,0}))+\sum_{k=0}^{N-1}\frac{1}{\sigma }t_{k}^{2}{\left(\sum_{i=1}^{m}L_{f_{i}}\right)}^{2}\left({\left(\sum_{i=1}^{m}\frac{1}{p_{i}^{2}}\right)}^{2}+1\right). \end{array}$$

Since $d_{H}(y,x_{N},y_{0,N})\geq 0$, as $y_{0,N}\in \partial H(x_{N})$, we get

$$\begin{array}{rl} & E\left(\min_{0\leq k\leq N-1}\sum_{i=1}^{m}f_{i}(x_{k})-\sum_{i=1}^{m}f_{i}(y)\right)\\ & \;\leq \frac{d_{H}(y,x_{0},y_{0,0})+\frac{1}{\sigma }{\left(\sum_{i=1}^{m}L_{f_{i}}\right)}^{2}\left({\left(\sum_{i=1}^{m}\frac{1}{p_{i}^{2}}\right)}^{2}+1\right)\sum_{k=0}^{N-1}t_{k}^{2}}{\sum_{k=0}^{N-1}t_{k}} \end{array}$$

and this finishes the proof.

Remark 3.4 The set from which the variable *y* is chosen in the previous theorem might seem to be restrictive, however, we would like to recall that in many applications domH is the set of feasible solutions of the optimization problem (3). Since $\text{im}(\nabla H^{*})=\mathrm{dom}\;\partial H:=\{x\in R^{n}:\partial H(x)\neq \emptyset \}\subseteq \mathrm{dom}\;H$, the inequality in Theorem 3.3 is fulfilled for every $y\in \text{im}(\nabla H^{*})$.

Remark 3.5 Note furthermore that so far we have not made any assumptions about the stepsizes in Theorem 3.3. It is, however, clear from the statement that in the case where $y=x^{*}$ for an optimal solution $x^{*}$ and the stepsizes $(t_{k}{)}_{k\in N}$ fulfil the classical condition that $\sum_{k=1}^{\infty }t_{k}=+\infty$ and $\sum_{k=1}^{\infty }t_{k}^{2}<+\infty$ it follows that $\lim_{N\in N}E(\min_{0\leq k\leq N-1}\sum_{i=1}^{m}f_{i}(x_{k})-\sum_{i=1}^{m}f_{i}(x^{*}))=0$.

The optimal stepsize choice, which we provide in the following corollary, is a consequence of [8, Proposition 4.1], which states that the function

$$z\mapsto \frac{c+(2\sigma {)}^{-1}z^{T}Dz}{b^{T}z},$$

where $c>0,b\in R_{++}^{d}:=\{(z_{1},\ldots ,z_{d}{)}^{T}\in R^{d}:z_{i}>0,i=1,\ldots ,d\}$ and $D\in R^{d\times d}$ is a symmetric positive definite matrix, attains its minimum on $R_{++}^{d}$ at $z^{*}=\sqrt{\left(2c\sigma /b^{T}D^{-1}b\right)}D^{-1}b$ and this provides $\sqrt{2c/\sigma b^{T}D^{-1}b}$ as optimal objective value.

Corollary 3.6 In the setting of Problem 3.1, assume that the functions $f_{i}$ are $L_{f_{i}}$-Lipschitz continuous on $\mathrm{im}(\nabla H^{*})$ for i=1,…,m. Let $x^{*}\in \mathrm{dom}\;H$ be an optimal solution of (3) and $(x_{k}{)}_{k\geq 0}$ be a sequence generated by Algorithm 3.2 with optimal stepsize

$$t_{k}:=\frac{1}{\sum_{i=1}^{m}L_{f_{i}}}\sqrt{\frac{d_{H}(x^{*},x_{0},y_{0,0})}{{\left(\sum_{i=1}^{m}\frac{1}{p_{i}^{2}}\right)}^{2}+1}}\frac{1}{\sqrt{k}}\;\forall \;k\geq 0.$$

Then for every N≥1 it holds

$$E\left(\min_{0\leq k\leq N-1}\sum_{i=1}^{m}f_{i}(x_{k})-\sum_{i=1}^{m}f_{i}(x^{*})\right)\leq 2\left(\sum_{i=1}^{m}L_{f_{i}}\right)\sqrt{\frac{d_{H}(x^{*},x_{0},y_{0,0})\left({\left(\sum_{i=1}^{m}\frac{1}{p_{i}^{2}}\right)}^{2}+1\right)}{\sigma }}\frac{1}{\sqrt{N}}.$$

Remark 3.7 In the last step of the proof of Theorem 3.3, one could have chosen to use the following inequality

$$\left(\sum_{k=0}^{N-1}t_{k}\right)E\left(\sum_{i=1}^{m}f_{i}\left(\frac{\sum_{k=0}^{N-1}t_{k}x_{k}}{\sum_{k=0}^{N-1}t_{k}}\right)-\sum_{i=1}^{m}f_{i}(y)\right)\leq \sum_{k=0}^{N-1}t_{k}E\left(\sum_{i=1}^{m}f_{i}(x_{k})-\sum_{i=1}^{m}f_{i}(y)\right)$$

given by the convexity of $\sum_{i=1}^{m}f_{i}(\cdot )$ in order to prove convergence of the function values for the ergodic sequence ${\bar{x}}_{k}:=(1/\sum_{i=0}^{k}t_{i})\sum_{i=0}^{k}t_{i}x_{i}$ for all k≥0. This would lead for every N≥1 and every $y\in R^{n}$ to

$$E\left(\sum_{i=1}^{m}f_{i}({\bar{x}}_{N-1})-\sum_{i=1}^{m}f_{i}(y)\right)\leq \frac{d_{H}(y,x_{0},y_{0,0})+\frac{1}{\sigma }{\left(\sum_{i=1}^{m}L_{f_{i}}\right)}^{2}\left({\left(\sum_{i=1}^{m}\frac{1}{p_{i}^{2}}\right)}^{2}+1\right)\sum_{k=0}^{N-1}t_{k}^{2}}{\sum_{k=0}^{N-1}t_{k}}$$

and for the optimal stepsize choice from Corollary 3.6 to

$$E\left(\sum_{i=1}^{m}f_{i}({\bar{x}}_{N-1})-\sum_{i=1}^{m}f_{i}(y)\right)\leq 2\left(\sum_{i=1}^{m}L_{f_{i}}\right)\sqrt{\frac{d_{H}(x^{*},x_{0},y_{0,0})\left({\left(\sum_{i=1}^{m}\frac{1}{p_{i}^{2}}\right)}^{2}+1\right)}{\sigma }}\frac{1}{\sqrt{N}},$$

and might be beneficial, as it does not require the computation of objective function values, which are by our implicit assumption of *m* being large expensive to compute.

## 4. A stochastic incremental mirror descent algorithm with Bregman proximal step

In this section, we add another nonsmooth convex function to the objective function of the optimization problem (3) and provide an extension of Algorithm 3.2, which evaluates in particular the new summand by a proximal type step. However, this asks for supplementary differentiability assumption on the function inducing the mirror map.

Problem 4.1 Consider the optimization problem

(9) $$\min_{x\in C}\sum_{i=1}^{m}f_{i}(x)+g(x),$$

where $C\subseteq R^{n}$ is a nonempty, convex and closed set, for every i=1,…,m, the functions $f_{i}:R^{n}\to \bar{R}$ are proper and convex and $g:R^{n}\to \bar{R}$ is a proper, convex and lower semicontinuous function, and $H:R^{m}\to \bar{R}$ is a proper, *σ*-strongly convex and lower semicontinuous function such that $C=\bar{\mathrm{dom}\;H}$, *H* is continuously differentiable on int(domH), $\mathrm{im}(\nabla H^{*})\subseteq \mathrm{int}(\overset{m}{\underset{i=1}{⋂}}\mathrm{dom}\;f_{i})\cap \mathrm{int}(\mathrm{dom}\;H)$ and int(domH)∩domg≠∅.

For a proper, convex, lower semicontinuous function $h:R^{n}\to \bar{R}$ we define its *Bregman-proximal operator* with respect to the proper, *σ*-strongly convex and lower semicontinuous function $H:R^{n}\to \bar{R}$ as being

$${\mathrm{prox}}_{h}^{H}:\mathrm{dom}\;\nabla H\to R^{n},\;{\mathrm{prox}}_{h}^{H}(x):=\underset{u\in R^{n}}{\arg\;\min}\{h(u)+D_{H}(u,x)\}.$$

Due to the strong convexity of *H*, the Bregman-proximal operator is well defined. For $H=(\frac{1}{2})∥\cdot {∥}^{2}$ it coincides with the classical proximal operator.

We are now in the position to formulate the iterative scheme we would like to propose for solving (9). In case *g*=0, this algorithm gives exactly the incremental version of the iterative method in [8], actually suggested by the two authors in this paper.

Algorithm 4.2 Consider for some initial value $x_{0}\in \text{im}(\nabla H^{*})$ and sequence of positive stepsizes $(t_{k}{)}_{k\geq 0}$ the following iterative scheme:

$$(\forall \;k\geq 0)\;\left[\begin{array}{l} \psi_{0,k}=x_{k}\\ for\;i=1,\ldots ,m\\ \;\;\psi_{i,k}=\nabla H^{*}(\nabla H(\psi_{i-1,k})-\epsilon_{i,k}\frac{t_{k}}{p_{i}}f_{i}^{\prime }(\psi_{i-1,k}))\\ end\\ x_{k+1}={\mathrm{prox}}_{t_{k}g}^{H}(\psi_{m,k}), \end{array}\right.$$

where $\epsilon_{i,k}isa\{0,1\}$ valued random variable for every i=1,…,m and k≥0, such that $\epsilon_{i,k}$ is independent from $\psi_{i-1,k}$ and $P(\epsilon_{i,k}=1)=p_{i}$ for every i=1,…,m and k≥0.

Lemma 4.3 In the setting of Problem 4.1, Algorithm 4.2 is well defined.

Proof. As $\mathrm{im}(\nabla H^{*})\subseteq \mathrm{int}(\overset{m}{\underset{i=1}{⋂}}\mathrm{dom}\;f_{i})$, it follows for every i=2,…,m and every k≥0 immediately that $\psi_{i-1,k}\in \mathrm{int}\;\mathrm{dom}\;f_{i}$, thus a subgradient of $f_{i}$ at $\psi_{i-1,k}$ exists.

In what follows we prove that this is the case also for $\psi_{0,k}$, for every k≥0. To this aim, it is enough to show that $x_{k}\in \mathrm{im}(\nabla H^{*})$ for every k≥0. For *k*=0 this statement is true by the choice of the initial value. For every k≥0 we have that

$$0\in \partial (t_{k}g+H-\langle \nabla H(\psi_{m,k}),\cdot \rangle )(x_{k+1}),$$

which, according to int(domH)∩domg≠∅, is equivalent to

$$0\in t_{k}\partial g(x_{k+1})+\partial H(x_{k+1})-\nabla H(\psi_{m,k}).$$

Thus, $x_{k+1}\in \mathrm{dom}\;\partial H=\mathrm{im}(\nabla H^{*})$ for every k≥0 and this concludes the proof.

Example 4.4 Consider the case when *m*=1, $\epsilon_{1,k}=1$ for every k≥0 and $H(x)=\frac{1}{2}∥x{∥}^{2}$ for x∈C, while H(x)=+∞ for x∉C, where $C\subseteq R^{n}$ is a nonempty, convex and closed set. In this setting, $\nabla H^{*}$ is equal to the orthogonal projection $P_{C}$ onto the set *C*. Algorithm 4.2 yields the following iterative scheme, which basically minimizes the sum $f_{1}+g$ over the set *C*:

(10) $$(\forall \;k\geq 0)\;x_{k+1}={\mathrm{prox}}_{t_{k}g}^{H}(P_{C}(x_{k}-t_{k}f_{1}^{\prime }(x_{k}))).$$

The difficulty in Example 4.4, assuming that it is reasonably possible to project onto the set *C*, lies in evaluating ${\mathrm{prox}}_{t_{k}g}^{H}$, for every k≥0, as this itself is a constraint optimization problem

$${\mathrm{prox}}_{t_{k}g}^{H}(x)=\underset{u\in C}{\arg\;\min}\left\{t_{k}g(u)+\frac{1}{2}∥x-u{∥}^{2}\right\}.$$

When $C=R^{n}$, the iterative scheme (10) becomes the proximal subgradient algorithm investigated in [15].

Theorem 4.5 In the setting of Problem 4.1, assume that the functions $f_{i}$ are $L_{f_{i}}$-Lipschitz continuous on $\text{im}(\nabla H^{*})$ for i=1,…,m. Let $(x_{k}{)}_{k\geq 0}$ be a sequence generated by Algorithm 4.2. Then for every N≥1 and every $y\in R^{n}$ it holds

$$\begin{array}{rl} & E\left(\min_{0\leq k\leq N-1}\left(\sum_{i=1}^{m}f_{i}+g\right)(x_{k+1})-\left(\sum_{i=1}^{m}f_{i}+g\right)(y)\right)\\ & \;\leq \frac{2\sigma D_{H}(y,x_{0})+\left(2{\left(\sum_{i=1}^{m}\frac{1}{p_{i}^{2}}\right)}^{1/2}+3+2m\right){\left(\sum_{i=1}^{m}L_{f_{i}}\right)}^{2}\sum_{k=0}^{N-1}t_{k}^{2}}{2\sigma \sum_{k=0}^{N-1}t_{k}}. \end{array}$$

Proof. Let $y\in \overset{m}{\underset{i=1}{⋂}}\mathrm{dom}\;f_{i}\cap \mathrm{dom}\;g\cap \mathrm{dom}\;H$ be fixed. For *y* outside this set the conclusion follows automatically.

As in the first part of the proof of Theorem 3.3, we obtain instead of (8) the following inequality which holds for every i=1,…,m and every k≥0

(11) $$\begin{array}{rl} E(D_{H}(y,\psi_{m,k})) & \leq E(D_{H}(y,x_{k}))+t_{k}E\left(\sum_{i=1}^{m}f_{i}(y)-\sum_{i=1}^{m}f_{i}(x_{k})\right)\\ & \;+\frac{1}{\sigma }t_{k}^{2}{\left(\sum_{i=1}^{m}L_{f_{i}}\right)}^{2}\left({\left(\sum_{i=1}^{m}\frac{1}{p_{i}^{2}}\right)}^{1/2}+1\right)-E\left(\sum_{i=1}^{m}\frac{1}{2}D_{H}(\psi_{i,k},\psi_{i-1,k})\right). \end{array}$$

As pointed out in the proof of Lemma 4.3, for every k≥0 we have

$$0\in t_{k}\partial g(x_{k+1})+\nabla H(x_{k+1})-\nabla H(\psi_{m,k}),$$

thus

$$t_{k}(g(y)-g(x_{k+1}))\geq \langle \nabla H(\psi_{m,k})-\nabla H(x_{k+1}),y-x_{k+1}\rangle .$$

The three point identity leads to

$$t_{k}(g(y)-g(x_{k+1}))\geq -(D_{H}(y,\psi_{m,k})-D_{H}(y,x_{k+1})-D_{H}(x_{k+1},\psi_{m,k}))$$

or, equivalently,

$$t_{k}(g(x_{k+1})-g(y))+D_{H}(y,x_{k+1})\leq D_{H}(y,\psi_{m,k})-D_{H}(x_{k+1},\psi_{m,k})$$

for every k≥0. Since the involved functions are measurable, we can take the expected value on both sides and obtain for every k≥0

(12) $$t_{k}E((g(x_{k+1})-g(y)))+E(D_{H}(y,x_{k+1}))\leq E(D_{H}(y,\psi_{m,k}))-E(D_{H}(x_{k+1},\psi_{m,k})).$$

Combining (11) and (12) gives for every k≥0

$$\begin{array}{rl} & t_{k}E((g(x_{k+1})-g(y)))+t_{k}E\left(\sum_{i=1}^{m}f_{i}(x_{k})-\sum_{i=1}^{m}f_{i}(y)\right)+E(D_{H}(y,x_{k+1}))\\ & \;\leq E(D_{H}(y,x_{k}))+\frac{1}{\sigma }t_{k}^{2}{\left(\sum_{i=1}^{m}L_{f_{i}}\right)}^{2}\left({\left(\sum_{i=1}^{m}\frac{1}{p_{i}^{2}}\right)}^{1/2}+1\right)\\ & \;-E(D_{H}(x_{k+1},\psi_{m,k}))-\sum_{i=1}^{m}\frac{1}{2}E(D_{H}(\psi_{i,k},\psi_{i-1,k})). \end{array}$$

By adding and subtracting $E(\sum_{i=1}^{m}f_{i}(x_{k+1}))$ and by using afterwards the Lipschitz continuity of $\sum_{i=1}^{m}f_{i}$, we get for every k≥0

$$\begin{array}{rl} & t_{k}E\left(\left(\sum_{i=1}^{m}f_{i}+g\right)(x_{k+1})-\left(\sum_{i=1}^{m}f_{i}+g\right)(y)\right)\\ & \;-t_{k}\left(\sum_{i=1}^{m}L_{f_{i}}\right)E(∥x_{k}-x_{k+1}∥)+E(D_{H}(y,x_{k+1}))\\ & \;\leq E(D_{H}(y,x_{k}))+\frac{1}{\sigma }t_{k}^{2}{\left(\sum_{i=1}^{m}L_{f_{i}}\right)}^{2}\left({\left(\sum_{i=1}^{m}\frac{1}{p_{i}^{2}}\right)}^{1/2}+1\right)\\ & \;-E(D_{H}(x_{k+1},\psi_{m,k}))-\sum_{i=1}^{m}\frac{1}{2}E(D_{H}(\psi_{i,k},\psi_{i-1,k})). \end{array}$$

By the triangle inequality, we obtain for every k≥0

$$\begin{array}{rl} & t_{k}E\left(\left(\sum_{i=1}^{m}f_{i}+g\right)(x_{k+1})-\left(\sum_{i=1}^{m}f_{i}+g\right)(y)\right)+E(D_{H}(y,x_{k+1}))\\ & \;\leq E(D_{H}(y,x_{k}))+\frac{1}{\sigma }t_{k}^{2}{\left(\sum_{i=1}^{m}L_{f_{i}}\right)}^{2}\left({\left(\sum_{i=1}^{m}\frac{1}{p_{i}^{2}}\right)}^{1/2}+1\right)-E(D_{H}(x_{k+1},\psi_{m,k}))\\ & \;+t_{k}\left(\sum_{i=1}^{m}L_{f_{i}}\right)E(∥x_{k}-\psi_{m,k}∥)+t_{k}\left(\sum_{i=1}^{m}L_{f_{i}}\right)E(∥\psi_{m,k}-x_{k+1}∥)\\ & \;-\sum_{i=1}^{m}\frac{1}{2}E(D_{H}(\psi_{i,k},\psi_{i-1,k})), \end{array}$$

which, due to Young's inequality and the strong convexity of *H*, leads to

$$\begin{array}{rl} & t_{k}E\left(\left(\sum_{i=1}^{m}f_{i}+g\right)(x_{k+1})-\left(\sum_{i=1}^{m}f_{i}+g\right)(y)\right)+E(D_{H}(y,x_{k+1}))\\ & \;\leq E(D_{H}(y,x_{k}))+\frac{1}{\sigma }t_{k}^{2}{\left(\sum_{i=1}^{m}L_{f_{i}}\right)}^{2}\left({\left(\sum_{i=1}^{m}\frac{1}{p_{i}^{2}}\right)}^{1/2}+1\right)-E(D_{H}(x_{k+1},\psi_{m,k}))\\ & \;+t_{k}\left(\sum_{i=1}^{m}L_{f_{i}}\right)E(∥x_{k}-\psi_{m,k}∥)+\frac{1}{2\sigma }t_{k}^{2}{\left(\sum_{i=1}^{m}L_{f_{i}}\right)}^{2}\\ & \;+E(D_{H}(x_{k+1},\psi_{m,k}))-\sum_{i=1}^{m}\frac{1}{2}E(D_{H}(\psi_{i,k},\psi_{i-1,k})). \end{array}$$

Since

$$∥x_{k}-\psi_{m,k}∥=∥\sum_{i=1}^{m}(\psi_{i-1,k}-\psi_{i,k})∥\leq \sum_{i=1}^{m}∥\psi_{i-1,k}-\psi_{i,k}∥,$$

we get for every k≥0 that

$$\begin{array}{rl} & t_{k}E\left(\left(\sum_{i=1}^{m}f_{i}+g\right)(x_{k+1})-\left(\sum_{i=1}^{m}f_{i}+g\right)(y)\right)+E(D_{H}(y,x_{k+1}))\\ & \;\leq E(D_{H}(y,x_{k}))+\frac{1}{2\sigma }t_{k}^{2}{\left(\sum_{i=1}^{m}L_{f_{i}}\right)}^{2}\left(2{\left(\sum_{i=1}^{m}\frac{1}{p_{i}^{2}}\right)}^{1/2}+3\right)\\ & \;+t_{k}\left(\sum_{i=1}^{m}L_{f_{i}}\right)E\left(\sum_{i=1}^{m}∥\psi_{i-1,k}-\psi_{i,k}∥\right)-\sum_{i=1}^{m}\frac{1}{2}E(D_{H}(\psi_{i,k},\psi_{i-1,k})). \end{array}$$

Young's inequality and the strong convexity of *H* imply that for every i=1,…,m and every k≥0

$$\begin{array}{rl} t_{k}\left(\sum_{i=1}^{m}L_{f_{i}}\right)∥\psi_{i-1,k}-\psi_{i,k}∥ & \leq \frac{1}{\sigma }t_{k}^{2}{\left(\sum_{i=1}^{m}L_{f_{i}}\right)}^{2}+\frac{\sigma }{4}∥\psi_{i-1,k}-\psi_{i,k}{∥}^{2}\\ & \leq \frac{1}{\sigma }t_{k}^{2}{\left(\sum_{i=1}^{m}L_{f_{i}}\right)}^{2}+\frac{1}{2}D_{H}(\psi_{i,k},\psi_{i-1,k}) \end{array}$$

and thus

$$\begin{array}{rl} & t_{k}E\left(\left(\sum_{i=1}^{m}f_{i}+g\right)(x_{k+1})-\left(\sum_{i=1}^{m}f_{i}+g\right)(y)\right)+E(D_{H}(y,x_{k+1}))\\ & \;\leq E(D_{H}(y,x_{k}))+\frac{1}{2\sigma }t_{k}^{2}{\left(\sum_{i=1}^{m}L_{f_{i}}\right)}^{2}\left(2{\left(\sum_{i=1}^{m}\frac{1}{p_{i}^{2}}\right)}^{1/2}+3+2m\right). \end{array}$$

Summing up this inequality from *k*=0 to *N*−1, for N≥1, we get

$$\begin{array}{rl} & \sum_{k=0}^{N-1}t_{k}E\left(\left(\sum_{i=1}^{m}f_{i}+g\right)(x_{k+1})-\left(\sum_{i=1}^{m}f_{i}+g\right)(y)\right)+E(D_{H}(y,x_{N}))\\ & \;\leq E(D_{H}(y,x_{0}))+\frac{1}{2\sigma }{\left(\sum_{i=1}^{m}L_{f_{i}}\right)}^{2}\left(2{\left(\sum_{i=1}^{m}\frac{1}{p_{i}^{2}}\right)}^{1/2}+3+2m\right)\sum_{k=0}^{N-1}t_{k}^{2}. \end{array}$$

This shows that

$$\begin{array}{rl} & E\left(\min_{0\leq k\leq N-1}\left(\sum_{i=1}^{m}f_{i}+g\right)(x_{k+1})-\left(\sum_{i=1}^{m}f_{i}+g\right)(y)\right)\\ & \;\leq \frac{2\sigma D_{H}(y,x_{0})+\left(2{\left(\sum_{i=1}^{m}\frac{1}{p_{i}^{2}}\right)}^{1/2}+3+2m\right){\left(\sum_{i=1}^{m}L_{f_{i}}\right)}^{2}\sum_{k=0}^{N-1}t_{k}^{2}}{2\sigma \sum_{k=0}^{N-1}t_{k}} \end{array}$$

and therefore finishes the proof.

The following result is again a consequence of [8, Proposition 4.1].

Corollary 4.6 In the setting Problem 4.1, assume that the functions $f_{i}$ are $L_{f_{i}}$-Lipschitz continuous on $\mathrm{im}(\nabla H^{*})$ for i=1,…,m. Let $x^{*}\in \mathrm{dom}\;H$ be an optimal solution of (9) and $(x_{k}{)}_{k\geq 0}$ be a sequence generated by Algorithm 4.2 with optimal stepsize

$$t_{k}:=\frac{1}{\sum_{i=1}^{m}L_{f_{i}}}\sqrt{\frac{2D_{H}(x^{*},x_{0})}{2{\left(\sum_{i=1}^{m}\frac{1}{p_{i}^{2}}\right)}^{2}+3+2m}}\frac{1}{\sqrt{k}}\;\forall \;k\geq 0.$$

Then for every N≥1 it holds

$$\begin{array}{rl} & E\left(\min_{0\leq k\leq N-1}\left(\sum_{i=1}^{m}f_{i}+g\right)(x_{k})-\left(\sum_{i=1}^{m}f_{i}+g\right)(x^{*})\right)\\ & \;\leq \left(\sum_{i=1}^{m}L_{f_{i}}\right)\sqrt{\frac{2D_{H}(x^{*},x_{0})\left(2{\left(\sum_{i=1}^{m}\frac{1}{p_{i}^{2}}\right)}^{2}+3+2m\right)}{\sigma }}\frac{1}{\sqrt{N}}. \end{array}$$

Remark 4.7 The same considerations as in Remark 3.7 about ergodic convergence are applicable also for the rates provided in Theorem 4.5 and Corollary 4.6.

Remark 4.8 Note the straightforward dependence of the optimal stepsizes as well as the right-hand side of the convergence statement on the data, i.e. the distance of the initial point to optimality, the Lipschitz constants $L_{f_{i}}$ and the probabilities $p_{i}$. This backs up the intuition that the decreased gradient evaluation, i.e. smaller $p_{i}$, does not come for free but at the cost of a worse constant in the convergence rate.

## 5. Applications

In the numerical experiments carried out in this section, we will compare three versions of the provided algorithms. First of all, the *non-incremental* version, which takes *full* subgradient steps with respect to the sum of all component functions instead of every single one individually. This can be viewed as a special case of the algorithms given, when *m*=1 and $\epsilon_{1,k}=1$ for all k≥0. Secondly, we discuss the *non-stochastic incremental* version, which uses the subgradient of every single component function in every iteration and thus corresponds to the case when $\epsilon_{i,k}=1$ for every *i*=1,...,*m* and every k≥0. Lastly, we apply the algorithms as intended by evaluating the subgradients of the respective component functions incrementally with a probability different from 1.

### 5.1. Tomography

This application can be found in [3] and arises in the reconstruction of images in PET. We consider the following problem

(13) $$\min_{x\in \Delta }-\sum_{i=1}^{m}y_{i}\log\left(\sum_{j=1}^{n}r_{ij}x_{j}\right),$$

where $\Delta :=\{x\in R^{n}:\sum_{j=1}^{n}x_{j}=1,x\geq 0\}$ and $r_{ij}$ denotes for i=1,…,m and j=1,…,n the entry of the matrix $R\in R^{m\times n}$ in the *i*-th row and *j*-th column and all of these are assumed to be strictly positive. Furthermore, $y_{i}$ denotes for i=1,…,m the positive number of photons measured in the *i*-th bin. As discussed in Example 2.5 this can be incorporated into our framework with the mirror map $H(x)=\sum_{i=1}^{n}x_{i}\log(x_{i})$ for x∈Δ and H(x)=+∞, otherwise. As initial value, we use the all ones vector divided by the dimension *n*.

We also want to point out that a similar example given in [8] in which the minimization of a convex function over the unit simplex Δ somehow does not match the assumption made throughout the paper as the interior of Δ is empty and the function *H* can therefore not be continuously differentiable in a classical sense. However, with the setting of Section 3 we are able to tackle this problem.

**Figure 1.**
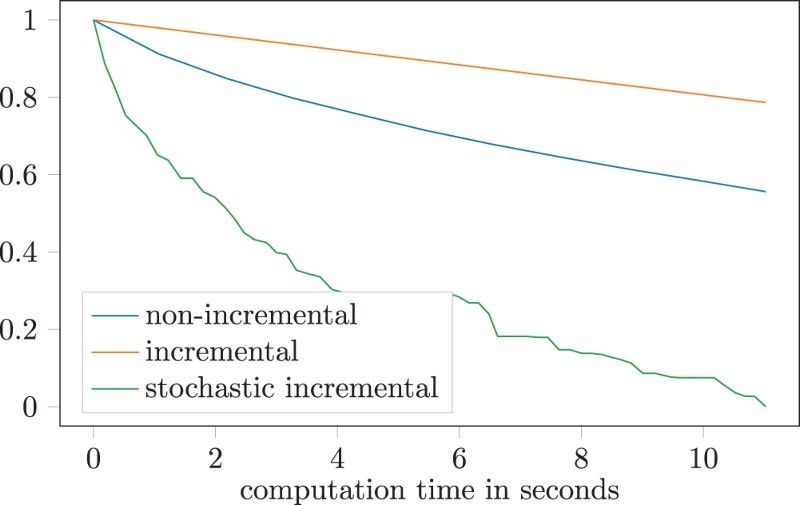
Results for the optimization problem (13). A plot of $\left(f_{N}-f(x_{best}))/(f(x_{0})-f(x_{best})\right)$, where $f_{N}:=\min_{0\leq k\leq N}f(x_{k})$, as a function of time, i.e. $x_{N}$ is the last iterate computed before a given point in time.

The bad performance, see Figure 1, of the deterministic incremental version of Algorithm 3.2 can be explained by the fact that many more evaluations of the mirror map are needed, which increases the overall computation time dramatically. The stochastic version, however, performs rather well, after only evaluating merely roughly a fifth of the total number of component functions, see Table 1.

**Table 1.** Results for the optimization problem (13), where NI denotes the non-incremental, DI the deterministic incremental and SI the stochastic incremental version of Algorithm 3.2.

		NI	DI	SI
$n=10^{4}$,	decrease obj.fun.val. (%)	0.066	0.032	0.15
*m*=3*n*	# outer loops	10	1	63
$p_{i}=0.003,\forall \;i$	# subgrad. evaluations	300,000	6939	6216
$n=10^{3}$,	decrease in obj.fun.val. (%)	0.196	0.515	0.671
*m*=6*n*	# outer loops	71	8	1769
$p_{i}=0.0016,\forall i$	# subgrad. evaluations	426,000	47,435	17,734

### 5.2. Support vector machines

**Figure 2.**
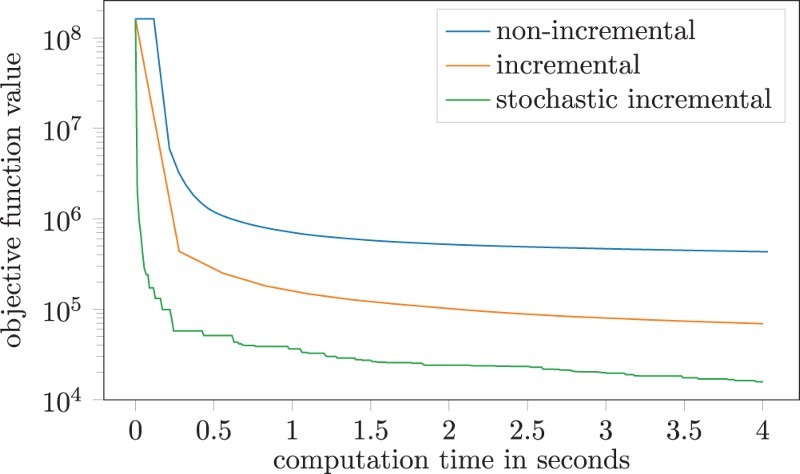
Numerical results for the optimization problem (14) with λ=0.01. The plot shows $\min_{0\leq k\leq N}f(x_{k})$ as a function of time, i.e. $x_{N}$ is the last iterate computed before a given point in time.

We deal with the classic machine learning problem of binary classification based on the well-known MNIST dataset, which contains 28 by 28 images of handwritten numbers on a grey-scale pixel map. For each of the digits, the dataset comprises around 6000 training images and roughly 1000 test images. In line with [4], we train a classifier to distinguish the numbers 6 and 7, by solving the following optimization problem

(14) $$\underset{w\in R^{784}}{\min}\sum_{i=1}^{m}\max\{0,1-y_{i}\langle w,x_{i}\rangle \}+\lambda ∥w{∥}_{1},$$

where for i=1,…,m, $x_{i}\in \{0,1,\ldots ,255{\}}^{784}$ denotes the *i*-th training image and $y_{i}\in \{-1,1\}$ denotes the label of the *i*-th training image. The 1-norm serves as a regularization term and λ>0 balances the two objectives of minimizing the classification error and reducing the 1-norm of the classifier *w*. To incorporate this problem into our framework, we set $H=\frac{1}{2}∥\cdot {∥}^{2}$ which leaves us with the identity as mirror map as this problem is unconstrained. The results comparing the three versions of Algorithm 4.2 discussed at the beginning of this section are illustrated in Figure 2. As initial value we simply use the all ones vector. All three versions show classical first-order behaviour, giving a fast decrease in objective function value first but then slowing down dramatically. More information about the performance can be seen in Table 2. All three algorithms result in a significant decrease in objective function after being run for only 4 s each. However, from a machine learning point of view, only the misclassification rate is of actual importance. In both regards, the stochastic incremental version clearly trumps the other two implementations. It is also interesting to note that it needs only a small fraction of the number of subgradient evaluations in comparison to the full non-incremental algorithm.

**Table 2.** Numerical results for the optimization problem (14), where NI denotes the non-incremental, DI the deterministic incremental and SI the stochastic incremental version of Algorithm 4.2.

		NI	DI	SI
λ=0.01	decrease in obj.fun.val. (%)	99.735	99.958	99.99
	#outer loops	83	16	370
	#subgrad. evaluations	999,006	179,531	36,962
	misclassified (%)	1.057	0.856	0.604
λ=0.001	decrease obj.fun.val. (%)	99.728	99.958	99.985
	#iter	75	15	336
	#subgrad. eval.	913,725	179,320	33,777
	misclassified (%)	1.007	0.856	0.403

Note: The computation for different regularization parameters *λ* show similar performances of the algorithms, but a lower misclassification rate for the lower value.

## 6. Conclusion

In this paper, we present two algorithms to solve nonsmooth convex optimization problems where the objective function is a sum of *many* functions which are evaluated by their respective subgradients under the implicit presence of a constraint set which is dealt with by a so-called *mirror map*. By allowing for a random selection of each component function to evaluate in each iteration, the proposed methods become suitable even for very large-scale problems. We prove a convergence order of $O(1/\sqrt{k})$ in expectation for the *k*th best objective function value, which is standard for subgradient methods. However, even for the case where all the objective functions are differentiable, it is not clear if better theoretical estimates can be achieved, due to the need of using diminishing stepsizes in order to obtain convergence in incremental algorithms. Future work could comprise the investigation of different stepsizes, such as constant or dynamic stepsizes as in [2]. Another possible extension of this would be to use different selection procedures such as random subsets of fixed size. Our framework, however, does not provide the right setting for such a *batch* approach as it would leave $\epsilon_{i,k}$ and $\epsilon_{j,k}$ dependent.
